# Protein Glycosylation Investigated by Mass Spectrometry: An Overview

**DOI:** 10.3390/cells9091986

**Published:** 2020-08-28

**Authors:** Anna Illiano, Gabriella Pinto, Chiara Melchiorre, Andrea Carpentieri, Vincenza Faraco, Angela Amoresano

**Affiliations:** 1 Department of Chemical Sciences, University of Naples Federico II, Via Cinthia 26, 80126 Napoles, Italy; anna.illiano@unina.it (A.I.); gabriella.pinto@unina.it (G.P.); chiara.melchiorre@unina.it (C.M.); acarpent@unina.it (A.C.); angamor@unina.it (A.A.); 2 CEINGE Advanced Biotechnology, University of Naples Federico II, Via Cinthia 26, 80126 Napoles, Italy; 3 Istituto Nazionale Biostrutture e Biosistemi—Consorzio Interuniversitario, Viale delle Medaglie d’Oro, 305, 00136 Rome, Italy

**Keywords:** glycosylation, post-translational modifications (PTM), mass spectrometry, quantitative analysis, glycosylation and diseases correlation

## Abstract

The protein glycosylation is a post-translational modification of crucial importance for its involvement in molecular recognition, protein trafficking, regulation, and inflammation. Indeed, abnormalities in protein glycosylation are correlated with several disease states such as cancer, inflammatory diseases, and congenial disorders. The understanding of cellular mechanisms through the elucidation of glycan composition encourages researchers to find analytical solutions for their detection. Actually, the multiplicity and diversity of glycan structures bond to the proteins, the variations in polarity of the individual saccharide residues, and the poor ionization efficiencies make their detection much trickier than other kinds of biopolymers. An overview of the most prominent techniques based on mass spectrometry (MS) for protein glycosylation (glycoproteomics) studies is here presented. The tricks and pre-treatments of samples are discussed as a crucial step prodromal to the MS analysis to improve the glycan ionization efficiency. Therefore, the different instrumental MS mode is also explored for the qualitative and quantitative analysis of glycopeptides and the glycans structural composition, thus contributing to the elucidation of biological mechanisms.

## 1. Glycosylation in Human Cells

Glycosylation is a co- and post-translational modification that involves the covalent bonding of an oligosaccharide chain with a polypeptide chain [1]. Glycans are secondary gene products generated by the coordinated action of many enzymes in the subcellular compartments of a cell. Therefore, monosaccharide units can be coupled together in many different ways (adopting different conformations and binding positions) without following a specific pattern, unlike proteins or DNA. Mammalian glycans are synthesized in intricate biosynthetic pathways by assembling only ten monosaccharide units: fucose (Fuc), galactose (Gal), glucose (Glc), *N*-acetylgalactosamine (GalNAc), *N*-acetylglucosamine (GlcNAc), glucuronic acid (GLCA), iduronic acid (IdoA), mannose (Man), sialic acid (SA), and xylose (Xyl) [2,3,4]. *N*- and *O*-linked glycosylation are the most common forms of glycosylated conjugate present in cells. To fully understand the origin of the diversity of glycan structures, it is necessary to deepen their biosynthetic transformations [5,6]. The synthesis of *N*-glycans begins in the endoplasmic reticulum (ER), where a lipid-bound oligosaccharide (which generally has a motif such as Glc3Man9GlcNAc2) is transferred “en bloc” to the Asn-X- (Ser/Thr) acceptor where X must be different from a proline residue, a consensus sequence specifically recognized by oligosaccharyltransferase (OST) [5]. After the removal of the two terminal glucose residues, the polypeptide associated with GlcMan9GlcNAc2 undergoes chaperone-guided folding, a series of transformations that are part of the protein quality control system [6]. The correctly folded proteins are then transferred to the different sections of Golgi apparatus that make the *N*-glycans chain elongated of fucosyl residues in *medial*-Golgi or of other monomers for their differentiation in hybrid type or complex-type in *trans*-Golgi. Finally, the ER machinery produces the stem region of the *N*-glycan while the *cis*-Golgi is the location of initiation of *O*-glycan biosynthesis. Glycan structural heterogeneity is a result of the non-template driven synthesis in the Golgi, reflecting the inefficiency in the initial transfer of glycans to proteins during the biosynthetic pathway. This phenomenon generates a complex mixture of glycosylated variants called *glycoforms*. It has been estimated that approximately 700 proteins and more than 7000 different structures are required to generate the full diversity of glycans in mammals [4].

## 2. Glycosylation in Other Organisms

The oligosaccharide component of the surface is specific to the type of cell and is altered when the cell undergoes evolutionary, physiological, and pathological changes. Oligosaccharides are often conjugated with lipids and/or proteins positioned on the cell membrane and are involved in intercellular communication and recognition processes mediated by the same carbohydrates in all of cells. For a long time, protein glycosylation was believed to be an exclusive prerogative of eukaryotes, until its occurrence was discovered in all living organisms from prokaryotes to eukaryotes. Although protein glycosylation in prokaryotic organisms appears to be a rare event and mainly regulated by different machinery compared to eukaryotic cells, bacteria can express glycoprotein displaying much more structural variation than that observed in eukaryotes [7]. Unlike bacteria, viruses as obligate parasites need to glycosylate their own proteins for host-cell machinery. Indeed, most viruses hijack the *N*- and *O*-linked glycosylation pathways of the host-cells to glycosylate their proteins, except for a few cases, e.g., chloroviruses and mimiviruses, capable of coding themselves some of the enzymes involved in the glycosylation event [8,9].

Unlike other prokaryotes, archaeal glycosylation displays numerous similarities with that from bacteria to eukarya mainly for the enzymatic activity of monomeric oligosyltransferases OST or dolichol phosphate carrier. The OST-mediated N-glycosylation, recurrent in eukarya and archaea, is restricted to a limited range of bacteria; whereas OST-mediated *O*-glycosylation appears to be representative of bacteria [10]. Anyway, the unique peculiarity of Archaea *N*-glycosylation in the unusual dolichol lipid carriers or new sugars as glycan constituents as well as in the variability of N-glycan composition under different growth conditions has been greatly reviewed [11].

The functional role of *N*-glycans dictated from the specific folding of glycoproteins drives the evolutionary changes in the precise signaling of folding control in eukaryotes [12] so that *N*-glycan synthesis has been demonstrated to be conserved among eukaryotic lineages [13] while its processing or *O*-glycan biosynthesis is kingdom specific [14].

Although the general path of biosynthesis and maturation of N-glycan is preserved in plants, their structure has been reported as species-specific. Like for fungi, the plant machinery generally produces glycoproteins with dense mannose *N*-glycans, as well as complex *N*-complex oligosaccharides containing β-1,2 xylose, α-1,3 fucose, and GlcNAc or Lewis a-trisaccharide [Fucα1-4(Galβ1-3)GlcNAc-R] [15,16]. The main difference compared to mammalian glycoproteins is based on the presence of α1,3-fucose instead of α1,6-fucose at GlcNAc proximal to Asn [17]. This substitution makes α1,3-fucose not recognizable by PNGase-F normally used for mammals *N*-glycoproteins. Plants produce oligosaccharides with reduced complexity and diversity, for example, there are no branched and sialylated *N*-glycans. Based on the results on model plants such as *A. thaliana* [18,19], it has been hypothesized that although complex *N*-glycans are not essential for the development and reproduction of plants, some modifications of *N*-glycan complexes such as β1,2-Xyl, α1,3-Fuc, and Lewis type A structures, are conserved. This evidence has also been found in higher plants and in mosses such as *Physcomitrella patens* as reported by Fitchette et al. 1999; Wilson, Zeleny, et al. 2001; Viëtor et al. 2003 [20,21,22]. With regard to *O*-glycosylation, implicated in cell signaling, it is substantially different in plants. Mucin-type *O*-glycans have not been detected on plant proteins as well as the glycosyltransferases necessary for the initiation and elongation of these *O*-glycans have not been found in plant genomes.

Studies on the glycoforms structures have been conducted in plants by Taylor et al. 2012; Tryfona et al. 2012; highlighting that a residue of Gal can be transferred to Ser residues on specific proteins and arabinose chains, and that structurally complex arabinogalactans are present on the hydroxyproline residues of the proteins belonging to the cell wall [23,24,25].

The need to include the fungi into the relative kingdom originated from several similarity with Animalia one. Actually, the highly mannose glycans like plants are characterized by extensive repeating α1-6-linked units branched by short chains of α1-2- and α1-3-linked mannose structures [26]. The intense *O*-mannosylation characterized the *O*-glycosylated proteins displaying a higher variable in sugar components and the linkage type of glycans determining the multiple functions [27]. For instance, the catalytic activity of α-L-arabinofuranosidase of fungus *Pleurotus ostreatus* relied in *O*-glycosylation of S160 residue crucial for enzyme structural stability [28].

## 3. Glycosylation and Disease

The main aim of glycomics is to understand the structure, enzymatic and biological mechanisms of glycosylation as well as to compare biosystems under normal and pathological conditions [29] in order to figure out glycans alterations as possible biomarkers of a disease. The difficulties caused by microheterogeneity largely prevent a simple and direct approach to investigate the entire set of glycans from glycoproteins after their hydrolysis or by glycoconjugates, and over the years different protocols have been developed [30,31,32,33]. An important aspect in glycoforms micro-heterogenicity molecular structures comprehension relies in their possible different pharmacological profiles [4]. Saccharide motifs commonly found in mammalian are object of pharmaceutical studies for the synthesis of therapeutically significant glycoconjugates like glycosaminoglycans, glycoproteins, glycolipids, glycosylphosphatidylinositol-anchored proteins, glycosylated recombinant proteins, and the development of carbohydrate-based vaccines [34] and manufacturing glycoprotein pharmaceuticals [35,36]. A new discipline born in 1980, glycobiology, has focused on the structural and functional characterization of glycoproteins and glycoconjugates. Recently, glycobiology has evolved in glycomics regarding the investigation of the complete set of glycoconjugates and carbohydrates present in an organism.

**Figure 1:**
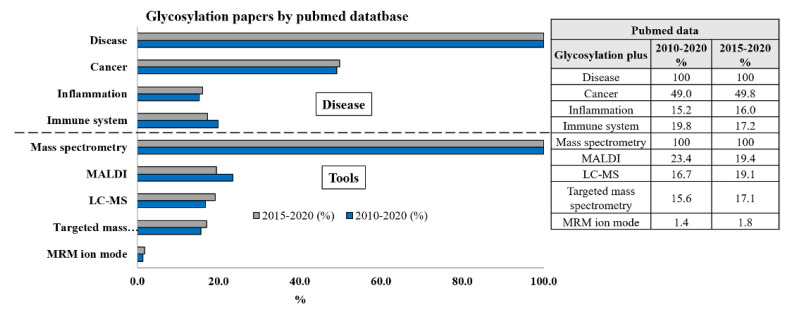
The number of papers calculated along a period of 5 to 10 years by the PubMed platform.

During the last five to ten years, many papers have been written on the role of glycosylation in different human diseases, whereas the highest contribution came out from the implication of glycosylation in cancer, followed by inflammation and involvement in the immune system (Figure 1).

The number of papers published during the last five years seems to be time-dependent, suggesting that the interest for the glycosylation in any disease is enormously increasing (Figure 1). What about the used technique along the time? Even the number of papers providing the use of the nuclear magnetic resonance (NMR) technique is maintained linear along that time frame, although it resulted to be almost 5-fold lower than those dealing with the mass spectrometry (MS) approach (data not reported in the Figure 1). A number of papers as high as 4863 and 2570 during the period 2010–2020 and 2015–2020, respectively, makes mass spectrometry a helpful tool for glycomics and matrix-assisted laser desorption/ionization mass spectrometry (MALDI-MS) or liquid chromatography tandem mass spectrometry (LC-MS/MS), equally contributing at the same aim (roughly 19% for both within last 5 years), whereas still a few papers referred to the targeted MS approaches) and even less to the LC-MS/MS in multiple reaction monitoring (MRM) ion mode (Figure 1).

An overview of each of these aspects will be discussed to support the crucial role of glycosylation and the relevance of new MS technologies in glycomics for the identification and quantification of the glycopeptides and the relative glycans.

### 3.1. Immunity and Inflammation

Immune system cells express glycoproteins and glycolipids associated with the cell surface able to detect environmental signals and modulate the adaptive immune response. A comprehensive review must deal with the antigenic role of glycoproteins, glycoconjugates, polysaccharides, or glycolipids as components of T-cell epitopes or toward their presentation by major histocompatibility complex (MHC) pathways on antigen-presenting cells [37]. Glycopeptides can bind to MHC molecules and to specifically stimulate T-cells thanks to their glycosylated portion, suggesting the structural importance of carbohydrate moiety for the T-cell stimulation and of the amino acid sequence to be allocated within a closed binding groove of MHC-I or MHC-II, in agreement with the length of peptide [38].

Many immune receptors expressed on innate and adaptive immune cells recognize the glycans present on the surface as molecular epitopes associated with pathogens (examples include bacterial lipopolysaccharides and peptidoglycans) [39]. Although protein glycosylation in prokaryotic organisms appears to be a rare event, regulated by a different machinery compared to eukaryotic cells, bacteria can express glycosylated protein [7]. Glycoproteins displayed on their surface are involved in pathogenicity, antigenicity, host–pathogen interactions, and immunity evasion as well as having structural functions [40,41]. For example, the alanine-proline-rich antigen (Apa) glycoprotein, expressed on the cell surface of different *Mycobacteria* species, induced glycan-specific T-cell response, whereas the non-glycosylated form of the same protein in *Escherichia coli* showed reduced stimulation of the CD4^+^ T-cell system compared to the native antigen, giving evidence of the crucial involvement of glycosylation in T-cell activation by Apa during infection [42]. A recent review greatly explored the different role of envelope glycoproteins along the virus pathobiology from immune evasion by glycan mimicry/shielding toward the recognition of glycans on host cell receptors up to induction of innate immune cell response mediated by complement activation [43]. Other authors lingered on the spike (S) envelope protein of the currently emerging virus (CoV) inducing severe acute respiratory syndrome (SARS) to explain the crucial role of glycoprotein in infection initiation by binding receptor-binding domain of S protein to the cellular receptor ACE2 and in the phase of viral envelope fusion with the host-cell membrane through the endosomal pathway [44]. Actually, the S proteins of coronaviruses display a larger number of *N*-linked glycan sites (23–38) per protomer but a lower population of oligomannose-type glycans (30%) compared to the other viruses [45]. The increase of the number of glycosylation sites and the reduced density by oligomannose-type glycans seems to be based on an evolutive selection, reflecting a balance between immune evasion by epitope shielding and functionality by attachment to the host cell. Although the S protein of SARS-CoV and SARS-CoV-2 showed ~91% identity with a high number of glycosylation sites shared between these two viral strains, the exclusivity of new sites of *N*-glycosylation in SARS-CoV-2 allowed to speculate a different shielding and camouflage from the host defense system [46]. The study of the different mechanisms of recognition based on the identification of glycosidic components of the virus by the immune system are fundamental for the development of antiviral vaccines [44,47,48].

Another important focus is the immune response by carbohydrates inducing the T cells to release cytokines in agreement to the activation mechanism [37]. In addition, known pro-inflammatory proteins, cytokines, can also induce direct changes to the N-glycosylation of endothelial cell membrane proteins, highlighting that glycosylation could contribute to the amplification of inflammatory vascular diseases [49]. A well-documented example is represented by the CD43 and CD45 glycoproteins abundantly expressed on the surface of B and T cells and which contain both *O*-glycans and *N*-glycans. During cell differentiation and activation, modulation of the glycosylation of these proteins is observed. This structural modification appears to be the molecular signal for the regulation of multiple T cell functions: cell migration, signaling of T cell receptors, cell survival, and apoptosis [50,51].

Aberrant glycosylation and/or alterations in serum protein glycosylation have been reported in many autoimmune and inflammatory diseases (e.g., rheumatoid arthritis, RA). The state of inflammation involves many physiological and biochemical systemic changes. Many studies have found that the mutation of the glycoside structure linked to a polypeptide chain reflected the pathophysiological state of the cell producing the protein. Therefore, knowledge of serum protein glycosylation can be an excellent starting point for the diagnosis and prognosis of many diseases [52,53].

The most representative example of the involvement of mutations in the saccharide structure in the onset of autoimmune and/or inflammatory diseases is given by immunoglobulin G (IgG). IgG is a glycoprotein with a N glycosylation site conserved in the Fc region and variable glycosylation (linked to O or N) in the Fab region [54]. Glycosylation of IgG molecules is essential for its binding with all receptors through the maintenance of an open conformation of the two heavy chains, whereas deglycosylated IgG antibodies are unable to mediate the inflammatory response triggered in vivo. A therapeutic application of gamma globulins intravenously indicates how these acts as anti-inflammatory [55].

Among all inflammatory conditions, rheumatoid arthritis is that in which IgG glycosylation has been studied mostly: decreased terminal sialylation and galactosylation of IgG is resulted to be the common denominator of autoimmune disorders [56]. The defect in glycosylation probably involves a greater interaction with the rheumatoid factor (RF), an autoantibody, which could contribute to increase the activation of cytokines and therefore the inflammatory response of the subject [57,58].

### 3.2. Genetic Defects and Cancer

Genetic defects in glycosylation (CDG) are often embryonic lethal, underlying the vital role of glycans in congenital defects affecting a single step in the formation of a glycoform or an entire pathway [59,60]. In fact, the CDGs were categorized into two different classes: type I concerns anomalies in the formation of the oligosaccharide structure on the glycolipid precursor before the attachment on the Asn residue of a protein; while type II concerns anomalies in the control of the branched oligosaccharide N-linked structure present on the new glycoprotein [61]. CDG phenotypes can result from altered activation or transport of sugar precursors; and altered expression and/or activity of enzymes (glycosidases or glycosyltransferases) or proteins implicated in the Golgi apparatus functioning. A clear illustration of congenital glycosylation disorder is the family of α-dystroglycanopathies whom frequently include alterations in the central nervous system [62] and ocular disease manifestations, in addition to muscular dystrophy, intellectual disability, developmental delay, hypotonia, macrocephaly, growth retardation, adducted thumbs, failure to thrive, cardiac anomalies, wrinkled skin, and early death. [39,63]

Genetic alterations support the development of various pathologies, including neoplastic ones, but epigenetic changes in response to stimuli can significantly influence the genesis and neoplastic transformation. The dynamic redistribution of proteins between subcellular compartments in response to cellular functional state has been clearly described as a consequence of system perturbation underlying breast cancer [64,65]. Even the glycosylation pathways are driven by the cellular response to microenvironmental cues that activate oncogenic pathways reprogramming cancer cells along the entire disease evolution up to invasion and disease dissemination [66]. Changes in protein glycosylation of both *O*-glycans (GalNAc-Ser/Thr) and *N*-glycans [67] can occur at the beginning as well as at end of cancer progression and metastasis. For instance, it has been shown that apparently minimal alterations in the structure of carbohydrates reflect the changes of cell surface components crucial for neoplastic transformations and the metastatic behavior of tumor cells [68,69].

Several reviews focus on the type of glycan alteration driving cancer hallmarks [66,70,71]:

(1) Glycan upregulation is a result of the cellular reprogramming likely due the metabolic shift from oxidative phosphorylation to aerobic glycolysis (Warburg effect) [72]. The increase of cellular glucose not only contributes to more sustained glycolysis but also to the upregulation of the hexosamine biosynthetic pathway. In parallel, the increase of β1-6 highly branched N-glycans observed in cancer cells [73] is also due to upregulation of N-acetylglucosaminyltransferase V enzyme (GnT-V encoded by *MGAT5* gene). Differently, GnT-III enzyme (encoded by *MGAT3* gene) contrasts *N*-glycans elongation by the addition of a bisecting GlcNAc residue in a β1,4-linkage. The overexpression of branched-*N*-glycan structures has been shown to interfere with cell–cell adhesion mediated by cadherin-epithelium, promoting dissociation and invasion of cancer cells.

(2) Incomplete biosynthesis of glycans can be due to the impairment of the normal synthetic mechanism of complex glycans or misfolding of proteins, triggering the end of biosynthesis. Truncated structures originated as a consequence of these events, often leading to the expression of Tn and T antigens, more commonly occurring in early carcinogenesis [74].

(3) The de novo expression of specific glycoepitopes e.g., certain antigens (such as sialyl Lewis a (SLe^a^) [Siaα2,3Galβ1,3(Fucα1,4)GlcNAc] and sialyl Lewis x (SLe^x^) [Siaα2,3Galβ1,4(Fucα1,3)GlcNAc]) is observed in advanced stages of cancer [75,76]. Lewis antigens originate from branched-N-glycan structures elongated with poly-N-acetyllactosamine (repeats of Galβ1,4 GlcNAcβ1,3) and further capped with fucose and sialic acid. These antigens are particularly enriched on the surface of cancer cells for high affinity to the carbohydrate-binding proteins, such as galectins and selectins. The interaction of glycans to both carbohydrate-binding proteins is a crucial event for neoplastic progression and the formation of cancer metastases [77,78,79]. An increased core fucosylation mediated by fucosyltransferase 8 is detected in metastatic melanoma [80], whereas the fucosyltransferase 8 expression could facilitate invasion and tumor dissemination, in part due to a reduced cleavage of the cell adhesion molecule L1 [81]. In each of these situations, the types of cell surface glycans present on a given glycoprotein are dictated in part by the expression, location, and activity of glycosyltransferases in a given cell [82].

(4) Other modifications on individual sugars, including O-acetylation of sialic acids and O-sulfation of galactose and N-acetylglucosamine residues can occur in cancer cells for modulating their growth and differentiation.

(5) New motifs e.g., Galβ1-4Galβ1- can take place within the complex-type oligosaccharide chain as an expression of dramatic changes in their biosynthesis during oncogenic transformation. These new-formed glycans are capped with α2-3-linked sialic acid residues demonstrated to facilitate the migratory behavior and to increase the invasiveness of metastatic melanoma cells [83].

The final glycosylation product reflects the coordinated effort of many different enzymes, whose expression, localization, and post-translational modifications are significantly influenced from cellular response of metabolic reprogramming [2]. As a result, reagents that specifically interact with the glycan product are crucial to manage the potential changes in glycosylation as an effective diagnostic, prognostic, and even therapeutic target in routine clinical practice.

Although the examination of changes in glycosylation in cancer lesions poses unique challenges, recent developments in glycomics offer promising solutions and may reveal specific associations between altered glycosylation and neoplastic or diseases progression. Technological breakthroughs in mass spectrometric analysis for specific glycan epitopes provide a more molecular approach to examine potential changes in glycosylation or to display a sufficient degree of alteration in glycosylation, as mixtures of commonly occurring glycosylation patterns associated with normal cells or tumor-associated signals [82,84].

A very interesting study concerns follicular melanoma (FL), the most common indolent B cell lymphoma, which represents about 40% of all non-Hodgkin lymphomas [85]. In this type of neoplasm, in about 85% of cases, variable domain glycans have been reported to be rich in high mannose structures [86,87]. The discovery of these structures in almost all patients implies that they are useful or even essential for improving proliferation and survival [88]. In healthy donors, these high mannose structures have not been detected on cell surface, advancing the hypothesis that in FL glycans do not fully mature within the Golgi complex due to enzyme inaccessibility [89,90].

All these changes of glycosylated chains contribute to increased molecular heterogeneity of tumor cells compared with their non-transformed counterparts, which in turn can alter the glycan structure and function. Therefore, the study of changes in the glycosylation mechanisms connected with the disease has become crucial to get information on the progression of cancer [91]. Such investigations constitute a breeding ground in considering glycans as important markers in the early diagnosis, in the determination of the prognosis and in the stratification of the risk, as well as in serving as a target of specific drug therapies [70].

## 4. Mass Spectrometry-Based Methodology in Glycoscience

### 4.1. Sample Preparation: Pre-MS Analysis for Glycoproteomics

The bottom-up proteomics studies provide the use of simple protocols of in solution or in situ digestion to release peptides from proteins to be submitted to the MS analysis. This approach enables the detection of a wide plethora of peptides mainly free from any variable modifications, such as phosphorylation or glycosylation, for the higher ionization efficiency in comparison to the modified peptides. Actually, an increase of the negative charge and the acidity of the phosphorylated or glycosylated peptides affects their ionization efficiency. This event clearly speculates that the MS signals of phosphorylated and glycosylated peptides undergo an effect of ion suppression due to the competition with a much higher number of non-modified peptide counterpart displaying a more intense ion current.

Many methods have been developed during the last decades to overcome the challenges associated to the glycoproteome analysis mainly based on mass spectrometry. The most widely used approach to characterize glycosylation involves the enzymatic or chemical cleavage of glycans from glycoprotein, followed by purification steps previous in the MS analysis. This method is limited exclusively to the glycoproteins with one glycosylation site, because it is unable to correlate glycan composition with the different glycosylation sites. This limitation can be overcome using another approach based on the characterization of intact glycopeptides released by glycoprotein proteolysis. Thus, the glycan composition can be correctly correlated to the glycosylation site of specific glycopeptides by using this approach referred to as ‘a glycosylation site-specific analysis’. The information obtained in a site-specific manner can be extremely important in correlating glycosylation profiles with specific glycosylation sites, which is useful in understanding structure–function relationships [92,93].

**Figure 2:**
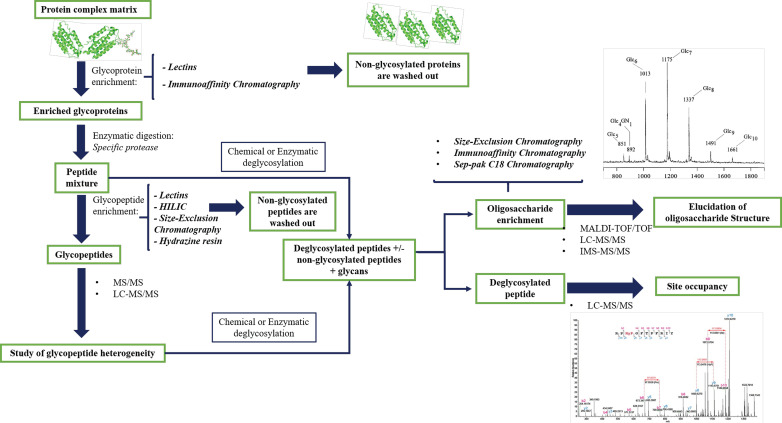
Workflow currently for mass spectrometry (MS)-based glycoproteomics approach.

Numerous protocols are now effective for the highly sensitive characterization of broad glycans by MS analysis [94]. Figure 2 summarizes the main stages of sample preparation combined with the MS analysis.

The most used protocols for biological samples provide the enrichment and purification of glycopeptides by using different molecular mechanisms. To overcome ionization difficulties and to prevent the suppression of the glycopeptide signal from the non-modified peptides in complex mixtures, it is possible to combine several purification methods. One of the most common methods for purifying glycopeptides before MS analysis is reverse phase purification (RP) on the high-pressure liquid chromatography (HPLC) system. This method provides a separation of the various glycopeptides based on their different amino acid sequence since the retention mechanism is governed by the hydrophobicity of peptide portion [95]. The techniques that could be combined with RP-HPLC include purification strategies based on hydrazine resins, lectin affinity chromatography, carbohydrate-based gels such as cellulose or sepharose, and gel filtration, or size exclusion chromatography (Figure 2).

A classical approach is based on the use of hydrazine-based resins to capture of N-linked glycoproteins. The oligosaccharides can be oxidized to the corresponding di-aldehydes and then immobilized on the solid hydrazide support. The resulting isolated glycoproteins are digested, and unmodified peptides washed out the resin. The glycopeptides are then enzymatically deglycosylated using PNGase (peptide-*N*-glycosidase) F (or A), an enzyme cleaving the bond between GlcNAc and an Asn residue converted to Asp, and quantified by isotopic labeling [96]. The oxidative chemical coupling between the glycan and resin is also the own limitation due to structural modification of glycans [97]. Additionally, chemical derivatization has several side reactions, therefore purification protocols based on lectin affinity chromatographic enrichment have recently been developed (Figure 2). A single lectin recognizes specific glycoforms, therefore lectins array could be used to capture several glycoproteins in a single step. Multi-lectin affinity columns have been developed by combining different lectins, e.g., Hancock and colleagues combine ConA (concanavaline A), WGA (wheat germ agglutinin) and Jacalin for the analysis of serum glycoproteins [98]. These enrichment methodologies have been used for comparative studies of human serum in pathological and non-pathological samples in order to identify oligosaccharides as disease biomarkers [98,99,100,101,102]. Alternative methods are based on the chemical and/or enzymatic hydrolysis of glycoproteins followed by multi-lectin affinity capture of the glycopeptides. This selective isolation of glycopeptides provides a great recovery of glycoforms because the steric hindrance by the protein portion seriously affects the interaction with lectin.

Other affinity purification techniques implemented in the last few years to purify glycopeptides are hydrophilic interaction chromatography (HILIC) or normal phase chromatography, and porous graphitized carbon. The porous graphitized carbon technique is used to enrich glycopeptides with small peptide portions, since not enough selectivity is obtained with larger tryptic glycopeptides. HILIC separation takes advantage of polar interactions between the hydroxy groups of glycans and the stationary phase. The efficient removal of the non-glycosylated counterpart takes place by using organic solvent washing followed by glycopeptide elution with an aqueous buffer as detailed in a study on the central nervous system glycoproteomics [103]. This method allows the glycopeptides separations based on their oligosaccharide portion, and its results are extremely useful when there is more than one glycosylation on the same peptide moiety. Among the main enrichment techniques, the use of immunoaffinity columns plays an important role in the characterization of site-specific occupancy due to the natural affinity of glycan epitopes to the specific antibodies on the functional regions. More often, the immunoaffinity is combined to another mentioned above protocol to reduce the complexity and increase the recovery of *N*-glycoproteins, for instance, in human plasma [104].

The further advantages and disadvantages associated with each used strategy is extensively reviewed in previous papers [92,93].

### 4.2. Glycans and Glycopeptides Characterization by MALDI-MS or LC-MS/MS

The analysis of glycans or glycopeptides has always been very challenging due to the limited quantities that are released from glycoproteins. Since the structure of a glycan may depend on the expression, activity, and accessibility of the different biosynthetic enzymes, it is not possible to use recombinant DNA technology in order to produce large quantities of glycans for structural and functional studies as it is for proteins. Mass spectrometry could be a valuable tool for their characterization thanks to high sensitivity and selectivity. MALDI-MS is an effective technique for *N*-glycan analysis of simple or complex matrices such as recently reported [105,106]. *Amoresano* et al. published a glycoproteomic characterization of human sera from healthy donors and patients affected by myocarditis for the identification of glycoproteins (even the least abundant), including the location of N-glycosylation sites and the profile of glycans present [107]. The strategy was simply based on the proteolytic digestion of serum proteins followed by a single enrichment step of glycopeptides by affinity chromatography using ConA lectin. The glycopeptides were then deglycosylated by treatment with PNGase-F and the free peptides analyzed by nano-LC/MSMS, which allowed both the identification of the individual proteins and the elucidation of their modification sites. Profiles of oligosaccharides released by MALDI-TOF (time of flight) were also obtained.

The glycans profile is obtained by MALDI-TOF analysis of the intact glycan mixture and the attribution of the different structures is carried out by checking the molecular weight and the knowledge of molecular pathways for the biosynthesis of oligosaccharides. However, this approach is useful in glycoforms profiling, but nevertheless it does not provide structural information such as sugar anomericity, neither on glycans site-specificity. To obtain this type of information, the combination of a profile by MALDI-TOF, with experiments of tandem mass spectrometry by post-source decay (PSD) or collision-induced dissociation (CID), is generally required [108]. The LC-MS/MS of whole glycopeptides provide, instead, more information about the site-specificity of glycans.

Usually the CID fragmentation of the glycopeptides produces a wide fragmentation on the oligosaccharide portion (such as typical oxonium ion fragment at *m*/*z* 163 [Hex + H]^+^, *m*/*z* 204 [HexNAc + H]^+^, *m*/*z* 292 [NeuAc + H]^+^, and *m*/*z* 366 [Hex-HexNAc + H]^+^ [105,106], and y-and b-type ions from the peptide moiety, therefore these MS/MS data are useful for assigning the glycan compositions (see Figure 4 below). In an analogous way, neutral losses of saccharides such as hexose (162 Da), N-acetylhexosamine (203 Da), fucose (146 Da), N-acetylneuraminic acid (291 Da) could be used to indicate the presence of glycopeptides in the mass spectra. Other types of fragments, called cross-rings, may be useful in determining the glycosidic linkage. MS^n^ experiments, on glycans moiety or directly on glycopeptides, are useful to characterize glycosidic structures present in glycoproteins as well as the type of branching, the sequence of the antennas, and the possible presence of modifying groups (e.g., sulfate, phosphate, acetyl groups, etc.). Moreover, by selecting fragments (typically oxonium ions) of the most abundant glycopeptides, it is possible to set up a selective ion monitoring (SIM) method for glycopeptides identification with high sensitivity in ion trap MS [107], quadrupole-TOF mass analyzers [108].

Another fragmentation method used in the analysis of glycopeptides are electron-capture dissociation (ECD) and electron transfer dissociation (ETD). In both techniques, the glycan portion does not undergo fragmentation while the peptide fragments provide both the z and c ions (see Figure 4 below). ECD experiments are typically performed on ionic resonance instruments of the Fourier transform cyclotron (FT-ICR) while the ETD can be performed in an ion trap mass spectrometer.

Finally, the combination of ion mobility-mass spectrometry (IM-MS) to the fragmentation induced by CID has been successfully used for improving the glycoform separation; indeed, the higher charged states associated to branched glycans generate repulsive interactions more intense than those less branched, leading to an improved separation between the different glycoforms on the same glycosylation site [109]. Many biomedical researches were based on the use of IM-MS to determine the profile of glycans in glycoproteins, as some specific glycans can change with disease progression [110]. Clemmer’s group showed that IM-MS was a promising technique to explore the glycan heterogeneity and glycan isomers [111] and to associate for the first time the ion mobility distributions of specific glycans to pathological conditions in liver cancer and cirrhosis patients [112].

### 4.3. Multiple Reaction Monitoring Targeted Mass Spectrometry Approach for Glycosylation Quantification

Structural characterization of complex carbohydrates is labor-intensive and time-consuming and requires orthogonal methods to identify: (i) the constituent monomers, (ii) their sequence including branching points, (iii) configuration of the glycosidic bond, (iv) position of the glycosidic bond, (iv) anomeric configurations [113,114]. In recent years, the high-throughput MS analyses of glycoforms have made significant progress and are now commonly applied [115,116]. Although MS has developed to be one of the most powerful technologies for the analysis of structures (e.g., protein sequencing, structural characterization of small organic molecules), it rarely allows to differentiate isobaric monosaccharide residues and to get information on the monosaccharide linkage, as mentioned above. Glycoproteins and their associated glycans quantification using MS techniques is at a nascent stage [117].

The improvements made in the sample preparation protocols and in the development of high-throughput platforms have led to an increase in the request of tools supporting data analysis in order to overcome the limitation of time expenditure [118,119,120]. To this aim, many software applications (open access and commercial) have emerged in recent years to offer new and promising capabilities as greatly reported in recent reviews [121,122,123,124]. Although these tools differ in the computational strategies, many of them have been designed to support specific workflows and/or limited data sets. As described by Liang et al., all these tools share the same fundamentals: (i) determining the accurate mass in the precursor MS spectra; (ii) hypothesis of glycan composition and the peptide backbone sequence from the analysis of the LC-MS/MS spectra; (iii) interrogation of theoretical spectra contained in databases of proteins and glycans that matches the marker fragment ions for the peptide backbone and glycan, or attempt to de-novo sequencing the portion of the glycan; and (iv) data validity score [124,125]. The use of such predictive software is particularly valuable for implementation of the LC-MS/MS method based on MRM, an ion mode widely recognized for the quantification accuracy of molecules. The main applications of MRM were limited to the metabolomics and proteomics, but their usefulness is also expanding in the field of glycoscience. MRM methods are very suitable for robust, fast, sensitive, and specific quantitative analysis of multiple target compounds, simultaneously detected also in the presence of other more abundant compounds (for instance, in complicated mixtures, such as biofluids).

Since MRM is a targeted approach, both the knowledge of the mass and charge state of the analyte and its fragmentation behavior in CID are essential for the choice of best transitions during the method development. Numerous studies have focused on the fragmentation model of glycans and glycopeptides [126,127,128], emphasizing that the typical fragmentation occurs on glycosidic bonds under low energy CID conditions normally used in triple quadrupole instruments. In these low energy conditions, the cross-ring cleavages are often low abundant, while intense fragments of glycan (oxonium ions) and those related to peptide fragmentation have been shown to be characteristic of each N-glycopeptides. Several authors performed glycoprotein quantification directly on the enzymatic digest of the plasma without any enrichment step in glycopeptides by using specific characteristic fragment ions [129,130,131]. As an example, an MRM/MS method was developed by Hong et al. to quantify immunoglobulins IgG, IgA, and IgM and their glycoforms by using the most intense signals associated to oxonium ion fragments for the quantification. On the other hand, in the case of O-glycosylation, the major fragment of the O-glycosidic bond is typically the Y0 fragment. These characteristic fragment ions are valuable to set up an MRM/MS method for glycoproteomics [132,133,134,135]. Characteristic oxonium ions, which represent hexose (*m*/*z* 163), HexNAc (*m*/*z* 204), NeuAc (*m*/*z* 292), HexHexNAc (*m*/*z* 366), HexHexNAc- Fuc (*m*/*z* 512), and HexHexNAcNeuAc (*m*/*z* 657), with or without loss of water, were used as reporter ions [136].

Moreover, due to the high variability of glycosylation, an important step in developing an MRM method for glycoproteins is the peptide and glycopeptide profiling (normally performed by LC-Q-TOF MS/MS) to evaluate the fragmentation behavior of the peptides and for validating the assignment of parent glycopeptide ion [137].

**Figure 3:**
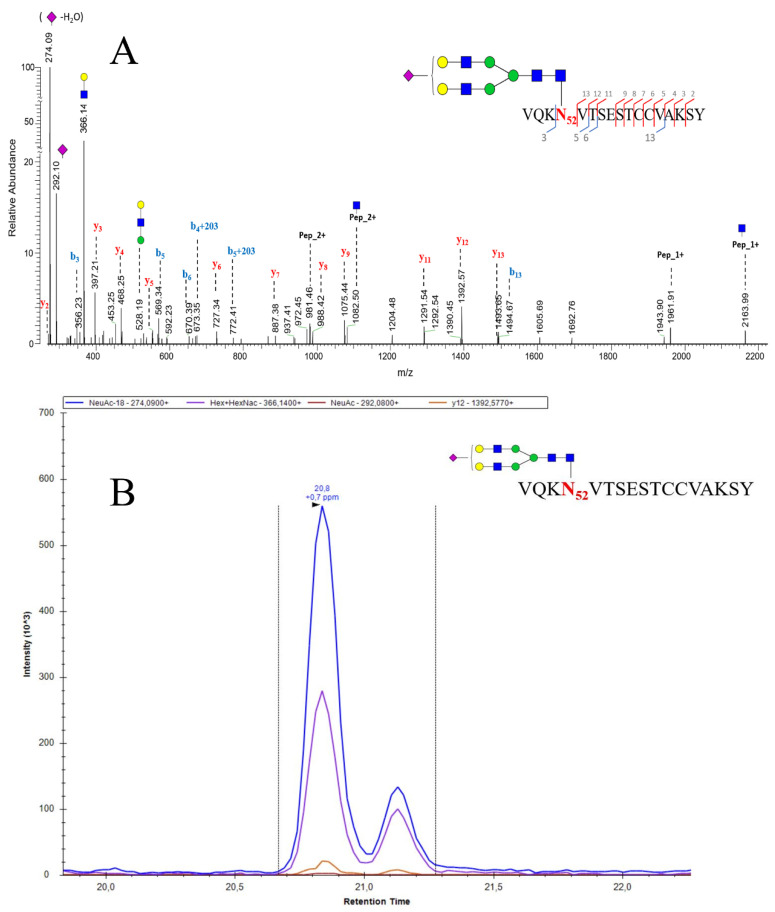
High-energy collision dissociation (HCD) fragmentation of the L.VQKN**_52_**VTSESTCCVAKSY.N glycopeptides with mono sialylated complex glycan at Asn_52_ (**A**) and EIC chromatogram (**B**) of the same glycopeptides (the two peaks are due to the different conformational isomers of the glycosidic structure [HexNac4Hex5NeuAc1]). 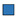 represents *N*-acetyl-glucosamine; 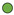 represents mannose; 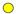 represents galactose; 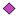 represents c sialic acid.

A glycosylation profile of standard (Sigma) pituitary human follicle stimulating hormone (hFSH) was performed by LC-MS/MS analysis using Q-Exactive plus spectrometer by Chiara Guerrera and coworkers (paper under revision). An example of high resolution MS^2^ fragmentation spectrum of hFSH alpha chian glycopeptide ([HexNac4Hex5NeuAc1] glycosylation on Asn52 site of L.VQKN_52_VTSESTCCVAKSY.N peptide) was reported in Figure 3A.

**Figure 4:**
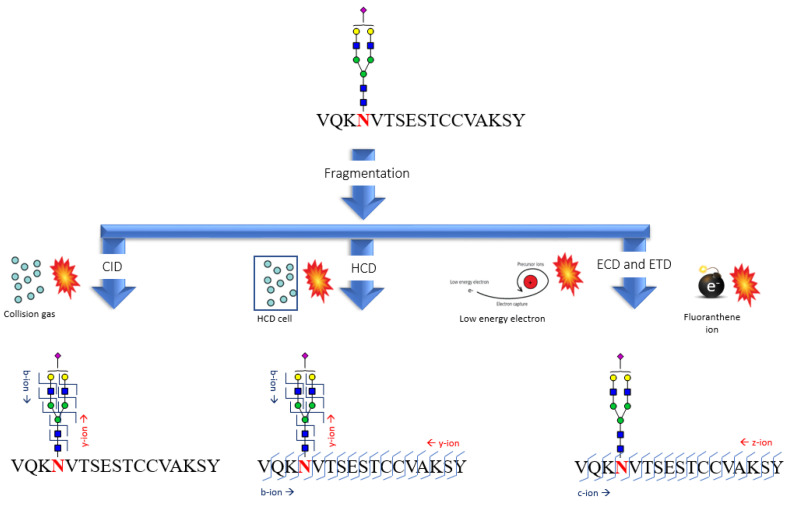
Mechanism of collision induced dissociation (CID), high-energy collision dissociation (HCD), electron-capture dissociation (ECD), and electron transfer dissociation (ETD) fragmentation. The glycopeptide shown in Figure 3A is used as example.

The fragmentation pattern of high-energy collision dissociation (HCD) is an alternative type of fragmentation method characterized by higher activation energy and shorter activation time comparing to the traditional ion trap CID (Figure 4). HCD for peptides with posttranslational modifications (PTMs) can provide both the sequence information (b- and y-type fragment ions) and the localization of the modification sites as it can identify CID-labile PTMs. Thus, high mass accuracy MS2 spectra have been successfully applied for PTMs studies, as certain diagnostic ions specific for HCD could be recognized for PTMs identification [138].

The MS/MS spectra offered a valid support for the choice of specific transitions to be used during the development of the quantitative MRM method. Actually, once established, the glycosylation pattern of the glycopeptide of interest, the most intense signals were selected to set up the MRM method. The extracted ion currents (EIC) associated to the different transitions for the same glycopeptide were recorded at the same retention time. This finding ensured the unequivocal identification above to contribute glycoprotein quantification. In Figure 3B, is shown the EIC best transitions of Figure 3A glycopeptide. Since the peptide’s glycan is a di-antennary mono-sialylated structure, this glycopeptide will exist as a combination of two isoforms generated by the position of the sialic acid on one or the other antenna and characterized by slightly different retention times (Figure 3B).

Therefore, transitions are representative of intense fragments ions that are unique for the peptide to be quantified, thus ensuring high sensitivity and reduced interference from other peptides [139,140].

Major complications can also arise in developing a glycopeptide quantification method because of the absence of exogenous glycopeptide standards and incomplete proteolytic digestion caused by steric hindrance due to the glycan chains [132,141,142].

As reported by Lebrilla et al., the application of MRM in the field of glycomics can be divided into three main areas: quantification of glycoproteins, glycopeptides, and oligosaccharides, showing each of them intrinsic troubles. In order to quantify glycoproteins, the crucial step is the selection of glycopeptides to be monitored [143]. Sample treatment protocols, as well as the choice of proteolytic enzymes, also largely influence the MRM analysis of both glycoproteins and glycopeptides [144,145]. Enzymatic hydrolysis is typically carried out with trypsin but, due to the uneven distribution of lysine and arginine residues in the amino acid sequences of proteins, this can generate high MW peptides not easily detectable by MS. Numerous advantages in this field have been obtained by using immunoaffinity enrichment of peptides or proteins followed by MRM/SRM-based quantification, achieving sensitivity suitable to the concentration range (ng/mL) at which low-abundance biomarkers are normally found [146,147,148]. Furthermore, in the analysis of glycopeptides, doubly glycosylated peptides can be generated, thus complicating the resolution of the glycosylation profile. Quantification strategies can be separated into label-based or label-free; standards with label or internal standards are often used in mass spectrometry to minimize the effects of ionization and to increase the accuracy of absolute and relative quantification. In particular, the type of labeling preferred in MRM analyzes is non-isobaric [149]. In order to overcome the limits of the large quadrupole inclusion windows (generally at least 0.5 Da), an alanine labeled with D^6^ has been used for the labeling of glycans, resulting in an Δm of 6 Da [150] or lysine and arginine for labeling at ^13^C and ^15^N of proteotypic peptides (Δm 8 and 10 Da). Anyway, in order to conduct a quantitative analysis of the glycoforms present in a mixture, it is essential to quantified non-glycosylated peptides in order to normalize the glycosylation profile to the total protein content [151].

Quantitative protein assays by using targeted MRM strategies have been developed to investigate protein concentrations in various biological fluids [152,153,154] e.g., in human plasma or serum, and other animal biofluids (e.g., bovine milk) [155,156,157].

Some examples of glycoproteins quantification based on mass spectrometry in MRM mode are annotated below to support the high potential and versatility of approach even in rapid routine clinical screening. Recently, a method for the quantification of total glycosylated and sialylated prostate-specific antigen (PSA) was recently developed. Periodate-oxidized PSA tryptic glycopeptides are captured using immobilized hydrazide, released by PNGase F, and quantified by MRM using a triple quadrupole LC-MS [158]. In a recent study by Song et al. [159], MRM assays were developed for the quantification in serum samples of fetuin and alpha1-acid glycoprotein glycopeptides. Kurogochi et al. developed an MRM assay to identify and quantify 25 glycopeptides from 16 different glycoproteins found in mice serum [135]. Moreover, MS in MRM mode has long been used for the quantitative determination of haptoglobin glycopeptides in the serum of psoriasis patients [160] or affected by pancreatic cancer [161]. Lebrilla et al. applied the LC-MS/MS methodology to investigate the glycoprotein profile of human milk by selecting the best transitions precursor ion-product ions for each glycopeptide.

## 5. Conclusions

The extensive literature on protein glycosylation reveals numerous examples in which these post-translational modifications play essential roles in the events of biological recognition, signaling and cell–cell communication. In fact, oligosaccharide structures perform crucial functions throughout the cell: in the cytosol, on the cell surface, in the secretory compartments, and in the extracellular space. Among the many roles that glycans play on the cell surface, the importance of specific glycosylated forms of the protein domain to facilitate or modulate biological recognition events has been highlighted. Therefore, the knowledge of the diversity of the structures of glycans becomes a further level of information content in the understanding of biological systems, laying the foundations for the greatest challenge of the near future, that is, identifying the critical contexts in which the functions of the glycans contribute to the biological regulation of the inside the surprisingly large array of heterogeneous glycan structures.

As widely described in this review, recent advances in qualitative and quantitative analytical strategies based on the use of mass spectrometry provide the necessary breadth, depth, and sensitivity of the analysis to define the entire spectrum of the complexity of glycan in various biological contexts. The continuous high-performance adaptations of these methodologies enable the collection of essential structural datasets to reveal the mechanisms in the biosynthetic pathway regulation, to define unique glycan signatures for pathological states and to provide correlations between structure and biological functions.

In this way, by increasing the ability to decode the numerous functions of glycoprotein or glycans in complex biological systems, the development of new therapeutic approaches, such as vaccines or targeted pharmacotherapies, is encouraged. Although technological progress in the field of targeted mass spectrometry has made great strides in the recognition of specific glycan structures, future studies are necessary for the realization of standardized methodologies that can be used in common clinical and diagnostic practice.

With the improvement and evolution of MS technology and sample preparation techniques, these types of test will play a more important role in the quantification of glycoproteins. In a futuristic scenario, to build high-performance platforms for the verification of cancer-exclusive glycoforms, these MRM-MS assays could be associated with methods to enrich robotic immunoaffinity [162,163].

## Aknowledgment

We thank Chiara Guerrera for providing assistance for LC-MS/MS analysis by using Q-Exactive plus spectrometer. This publication was in partial fulfilment of the requirements for the degree of PhD in Chemical Sciences−XXXIII Cycle, University of Naples Federico II.
